# Integrative Insight into Relationships between Florivorous Thrips *Haplothrips leucanthemi* and *H. niger* (Insecta, Thysanoptera)

**DOI:** 10.3390/insects13030279

**Published:** 2022-03-11

**Authors:** Agnieszka Kaczmarczyk-Ziemba, Halina Kucharczyk, Marek Kucharczyk, Kinga Kucharska

**Affiliations:** 1 Department of Evolutionary Genetics and Biosystematics, Faculty of Biology, University of Gdansk, 80-308 Gdansk, Poland; 2 Department of Zoology and Nature Protection, Faculty of Biology and Biotechnology, Maria Curie Sklodowska University, 20-033 Lublin, Poland; halina.kucharczyk@mail.umcs.pl (H.K.); marek.kucharczyk@mail.umcs.pl (M.K.); 3 Faculty of Biology and Biotechnology, Maria Curie Sklodowska University, 20-033 Lublin, Poland; kinga.stanislawek@gmail.com

**Keywords:** molecular markers, microbiota, *Haplothrips*, synonym, integrative approach

## Abstract

**Simple Summary:**

In this work, we aimed to determine the genetic diversity of *Haplothrips leucanthemi* and *H. niger*. The latter is recognized as a parthenogenetic form of *H. leucanthemi* and is also considered to be a pest in clover-seed plantations. Molecular analyses were performed at both the mitochondrial (COI) and nuclear levels (28S and ITS2). Additionally, as a part of an integrative approach, we determined and analyzed their microbiota profiles, based on high-throughput 16S rRNA gene sequencing.

**Abstract:**

*Haplothrips niger* is recognized as a parthenogenetic form of *H. leucanthemi* and is also considered to be a pest in clover-seed plantations. On the contrary, some researchers highlight the distinctiveness of *H. niger* and *H. leucanthemi*. Taking into account these two points of view, as well as the lack of molecular studies investigating the relationship between the mentioned thrips, we decided to perform analyses of both mitochondrial (COI) and nuclear markers (28S and ITS2) to determine the genetic diversity of *H. leucanthemi* and *H. niger*. Additionally, as a part of an integrative approach, we determined and analyzed their microbiota profiles, based on high-throughput 16S rRNA gene sequencing. The results of the molecular analyses revealed high intraspecific diversity of *H. leucanthemi* and did not support the distinctiveness of *H. niger*. The identified microbiota profiles were similar in both species and the performed analyses also did not support the distinctiveness of *H. niger*. Significant differences were, in turn, observed between *H. leucanthemi* and *H. niger* larvae. Moreover, two known endosymbiotic bacteria were found in the analyzed microbiota profiles (i.e., *Wolbachia* and *Rickettsia*). Nevertheless, these symbionts were not predominantly found in the bacterial communities that are associated with *H. niger* and thus, its impact on the parthenogenetic mode of its reproduction seems less likely.

## 1. Introduction

The systematics and taxonomy of the order Thysanoptera at the genus level have been under discussion for many years, and has been recently summarized by Mound and Hastenpflug-Vesmanis (2021) [1]. In this group of insects, synonymization is frequently noticed, not only at the genus level (currently, 420 names in synonymy) but also at the species level (see ThripsWiki 2021). Many thrips species that are currently considered to be synonymous have been described based only on their color, body size, or sexual and alary dimorphism [2,3]. Those issues have been recognized, e.g., for the genus *Haplothrips* (inter alia for *H. davisi* and *H. fissus*, *H. robustus* and *H. sesuvii*, or *H. tritici* and *H. cerealis* species pairs) [2,4,5,6]. Although *Haplothrips* is the third largest genus in Thysanoptera (240 species, [1]), there are very few studies that involved molecular analyses or evaluations of *Haplothrips* taxonomy or the determination of the genetic distances among the valid species. The study described by Timm et al. (2008) provided a COI-based molecular identification key for *H. nigricornis* and *H. clarisetis* and gave preliminary insight into the genetic distance between those two species [7]. More recently, a study based on DNA barcoding has been applied in order to evaluate the relationships between two morphospecies: *H. andresi* and *H. gowdeyi*. Although both mentioned species can be distinguished by morphological characteristics, the determined genetic distance between them is low, and thus not all of the applied delimitation methods indicate them as distinct species [8].

However, the molecular studies mentioned above involved *Haplothrips* species that are not synonymized. In the present study, we applied an integrative approach in order to determine relationships between florivorous *Haplothrips leucanthemi* (Schrank, 1781) and *H. niger* (Osborne, 1883), and to verify their potential distinctiveness. Currently, *H. niger* is considered a parthenogenetic synonym [4,5] but, on the contrary, the European literature refers to the distinctiveness of *H. leucanthemi* and *H. niger* [9]. Both species seem to be strictly associated with different host plants: *H. leucanthemi* with *Leucanthemum vulgare* and *L. ircutianum* (Asteraceae), and *H. niger* with *Trifolium* species (Fabaceae) [9], which suggests that *H. niger* can be recognized as an *H. leucanthemi* biotype. Importantly, *H. niger* is also considered to be a pest in clover-seed plantations, feeding both on flowers (crown petals or pollen) and fruits, which in turn reduces the seed yield [10,11,12]. Parthenogenetic *H. niger* females are more fertile, which may result in a rapid increase in its populations in infested plantations. In turn, the bisexual *H. leucanthemi* is not a recognized pest of *Trifolium* plants and its individuals are effective pollinators of Asteraceae plants. Hence, determining the relationships between those two species is important not only for the proper description of thrips-species diversity, but also for the identification of pests that threaten commercial crops.

**Figure A1:**
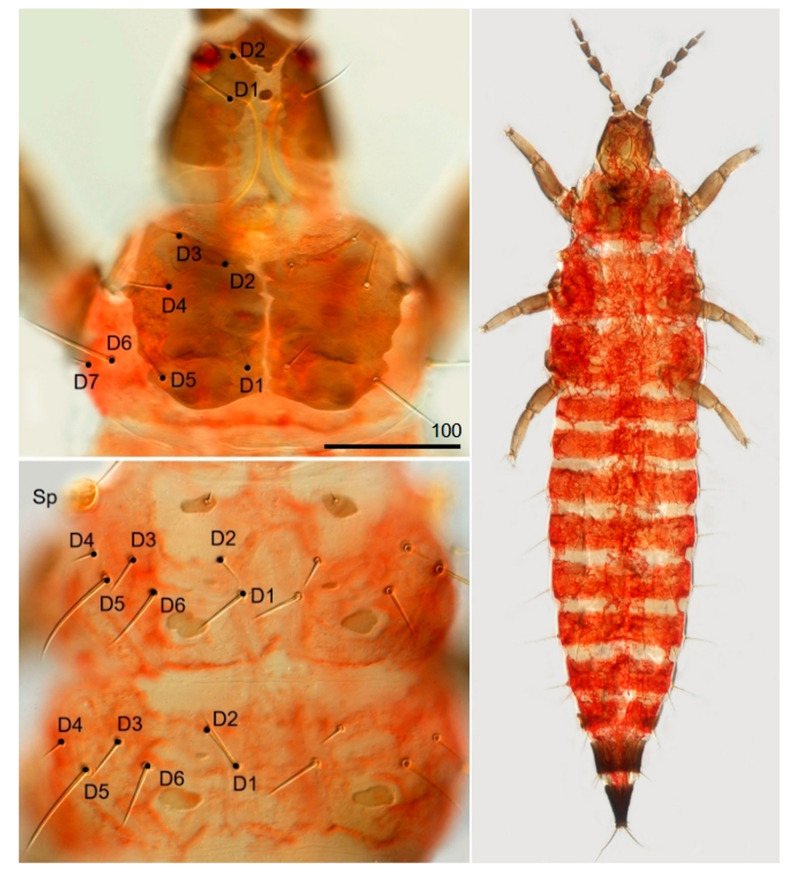
*H. leucanthemi*—second larval instar: body view (**right**), head and pronotum (**left**, **above**), meso- and metanotum (**left**, **below**), Sp—spiracle, D1–D7—dorsal setae on head and thorax included in morphometric measurements (Table 2).

**Figure A2:**
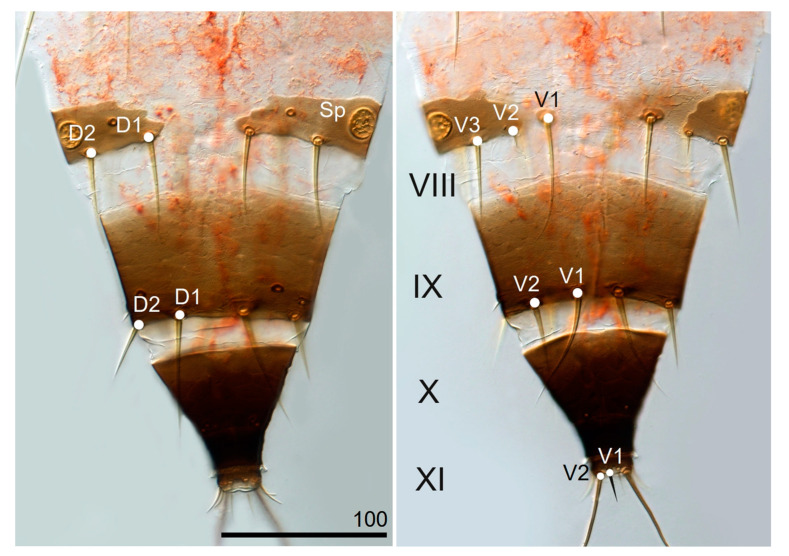
*H. leucanthemi*—second larval instar; abdominal segments VIII-XI: dorsal (**left**) and ventral (**right**) sites, Sp—spiracle, D1, D2, V1–V3—dorsal and ventral setae on abdomen included in morphometric measurements (Table 2).

**Figure A3:**
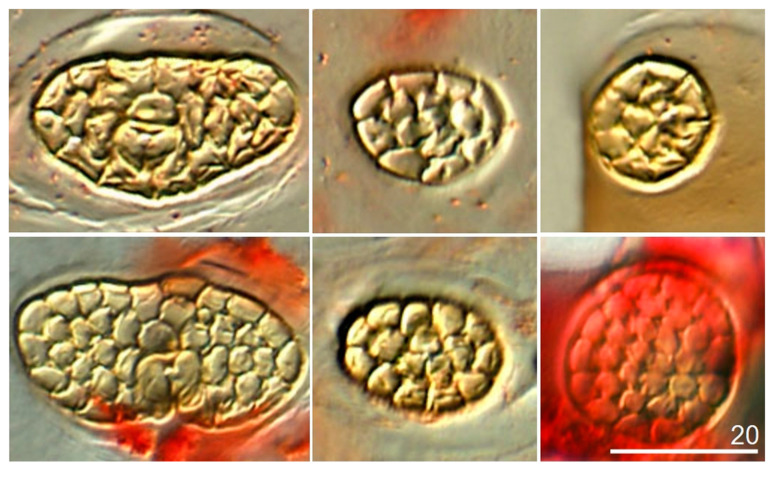
Structure of spiracles on mesothorax (**left**), second abdominal segment (**middle**) and eighth abdominal segment (**right**) in *H. niger* (**above**) and *H. leucanthemi* (**below**) second larval instar (Table 2).

Priesner (1964) [13], Schliephake and Klimt (1979) [14], and Moritz (2006) [15] described morphological differences between *H. leucanthemi* and *H. niger,* but further studies revealed that the measurements overlap considerably and the definitive separation of both species is problematic [9]. However, measurements performed by members of our team (H.K. and K.K.) on archival specimens revealed the existence of some subtle morphological differences in both adults and second instar larvae that might be species specific (details in Appendix A: Table A1 and Table A2, Figure A1, Figure A2 and Figure A3). First of all, in adults the prothorax and abdominal segment IX are longer in *H. leucanthemi*, but the prothorax epimeral setae are longer in *H. niger*. Differences can also be seen in the postocular setae, which are pointed at the apex in *H. leucanthemi* and blunt in *H. niger* adults. Similar observations have been previously described by Schliephake and Klimt (1979) [14], and Moritz (2006) [15]. Moreover, a new potentially species-specific difference was observed in adults: the spiracle on abdominal segment VIII is round and its diameter is larger in *H. leucanthemi* than in *H. niger* (17.5 μm versus 10 μm on average). Additional potentially species-specific differences have also been identified for second instar larvae. Measurements of the body, antennae, as well as the prothorax setae (D1, D4, D5, and D6) lengths revealed their higher values in *H. niger* larvae. In turn, the length and width of spiracles on the mesothorax and abdomen (segments II and VIII), and the numbers of cells in the spiracles were higher in *H. leucanthemi* larvae.

Taking into account those premises that prove at least the subtle distinctiveness of *H. leucanthemi* and *H. niger*, as well as the lack of molecular studies investigating their relationships, we decided to perform analyses of both mitochondrial (COI) and nuclear markers (28S and ITS2) to determine the genetic diversity of *H. leucanthemi* and *H. niger*. Additionally, as part of an integrative approach, we determined and analyzed the microbiota profiles associated with both *H. leucanthemi* and *H. niger*. Those analyses were based on high-throughput 16S rRNA gene sequencing.

## 2. Materials and Methods

*Haplothrips leucanthemi* and *H. niger* are not endangered or protected in the sampling areas, and sampling activities were not carried out at locations where specific permission is required. Specimens for molecular analyses were collected in June and July of 2019 from five sampling sites located in eastern Poland (Table 1). All larvae were collected from the same plants as adults. Species identification was performed based on the morphological traits described by Schliephake and Klimt (1979) [14], and Moritz (2006) [15]. The collected specimens were reared on their host plants: *Leucanthemum vulgare*, *L. ircutianum*, *Trifolium montanum*, and *T. pratense*, respectively. Thrips feeding in flowers of those plants were put into round plastic boxes (diameter 100 mm, height 40 mm) with a ring of moist filter paper placed on the bottom. All containers were stored in phytotron chambers under laboratory conditions at 22 °C with 16 h of light, 8 h of dark, and a 60% humidified atmosphere [16]. Both larvae and adults (females) were then picked from host plants, individually collected into sterile Eppendorf tubes and then stored at −80 °C until further analysis.

The collected specimens were then surface sterilized in 95% ethanol for 5 s to remove surface microbes and then washed three times in sterile distilled water. Finally, all individuals were separately transferred to sterile, distilled water, and then lysis buffer and proteinase K were added. DNA was extracted using Sherlock AX kit (A&A Biotechnology, Gdansk, Poland) according to the manufacturer’s protocol. Genetic material was extracted from the entire body of each of the collected thrips (specimens were not pooled). Sterile equipment was used to avoid cross-contamination of samples. The quantity and quality of the extracted DNA were evaluated using a NanoDrop ND-1000 spectrophotometer (NanoDrop Technologies Inc., Wilmington, DE, USA). After extraction, the DNA was stored at −20 °C until further use.

The mitochondrial COI fragment was amplified using primers HCO-2198/LCO-1490 [17]. Nuclear markers were amplified using THrd1a/28S B (28S) [18,19] and ITSF/ITSR (ITS2) [20] primers, respectively. In the case of 28S, additional internal primers were used during sequencing (28S THrd3a and 28S THrd3b, respectively) [19]. All PCR protocols are given in Table S1. Products of amplification were separated by 1% agarose gel electrophoresis in a 1x SB buffer and visualized with SimplySafe (EURx, Gdańsk, Poland) in UV light. All products were purified with EPPiC Fast (A&A Biotechnology, Gdynia, Poland) according to the protocol and sequenced with the BigDye terminator cycle sequencing method. In the case of ITS2, only amplicons obtained for homozygous specimens were sequenced. All newly obtained sequences were deposited in the GenBank database (accession nos. MZ047773-MZ047779 for COI, MZ191780-MZ191791 for 28S, and MZ478147-MZ478157 for ITS2).

The genetic material extracted from collected specimens was also used for microbiota profiling. The V3-V4 hypervariable regions of the bacterial 16S rRNA gene were amplified using primers 341F/785R [21]. Libraries were prepared with a two-step PCR protocol based on Illumina’s “16S metagenomic library prep guide” (15044223 Rev. B), NEBNext^®^ Q5 Hotstart High-Fidelity DNA polymerase (New England BioLabs Inc., Ipswich, MA, USA), according to the manufacturer’s protocol using Q5^®^ Hot Start High-Fidelity 2× Master Mix (NEBNext—New England BioLabs). The PCR conditions were as follows: 98 °C for 30 s for initial denaturation, 98 °C for 10 s, 55 °C for 30 s, 72 °C for 20 s repeated for 25 cycles, followed by a final extension at 72 °C for 2 min, and the Nextera Index kit (2 × 250 bp). Paired-end (PE, 2 × 250 nt) sequencing with a 5% PhiX spike-in was performed with an Illumina MiSeq (MiSeq Reagent kit v2) at Genomed, Warsaw, Poland, following the manufacturer’s run protocols (Illumina, Inc., San Diego, CA, USA). The automatic primary analysis and the demultiplexing of the raw reads were performed with MiSeq with the use of MiSeq Reporter (MSR) v2.6 (16S Metagenomics Protocol). Raw NGS data were deposited and are fully available in the European Nucleotide Archive (accession number PRJNA730011).

The obtained raw sequential reads were then analyzed using dedicated software. Mitochondrial and nuclear sequences were analyzed using the BLAST application (Basic Local Alignment Search Tool) to browse sequences deposited in the NCBI database and to identify sequences that were homologous with them [22]. All sequences were manually corrected using BIOEDIT 5.0.9 [23] and aligned in CLUSTAL OMEGA using default settings [24]. Additionally, COI sequences were translated into amino-acid sequences using the EMBOSS-TRANSEQ application to check against pseudogenes [25,26]. The software package DNASP 5.10.01 was used to retrieve haplotypes [27] and MEGA X software was applied in order to calculate the uncorrected *p*-distances [28]. Additionally, in MEGA X we calculated the uncorrected *p*-distances among other species belonging to the *Haplothrips* genus. Sequences submitted to the GenBank public database were used in that analysis and its aim was to estimate the range of interspecies genetic distance within the genus. Moreover, the COI sequences obtained here were combined with those from GenBank to reconstruct phylogenetic relationships among the selected *Haplothrips* species. An analysis in jMODELTEST 2.1.10 with the assumptions of the Bayesian information criterion [29] allowed us to determine a model of evolution that best fit the data (HKY + I + G). A phylogenetic analysis was carried out using the Bayesian approach implemented in MRBAYES 3.1.2 [30]. The Markov chain Monte Carlo search was run with four chains for 10 million generations with a sampling frequency of 1/1000 trees, with a burn-in of 10%. The sequence of *Haplothrips carpathicus* (GenBank accession number: MG491888) was used as an outgroup in that analysis. The final phylogenetic tree was visualized in FIGTREE 1.4.2 (http://tree.bio.ed.ac.uk/software/figtree/, accessed on 23 November 2021) and edited in INKSCAPE 1.0.1 (https://inkscape.org, accessed on 23 November 2021). In turn, the reconstruction of the phylogenetic relationships among the haplotypes identified in the present study, with a TCS method [31], was undertaken in POPART software [32]. Outgroup sequences were not added to those analyses.

In the case of the microbiota profiling, demultiplexed paired-end reads were imported into QIIME2 (2019.1 release) [33]. The DADA2 algorithm was applied to filter out noise and correct errors in marginal sequences, remove chimeric sequences, merge paired-end reads, and summarize amplicon sequence variants (ASVs) [34]. The taxonomy assignment was performed with a pre-trained SILVA 132 99% out-based Naïve-Bayes classifier [35]. ASVs matching with chloroplast and mitochondrial sequences were removed from the dataset for downstream analyses. The determination of alpha and beta diversity was performed using the QIIME2 core-diversity metrics. The dataset was rarefied to a depth of 21,193 reads per sample. Differences in alpha-diversity indices among the sample groups were statistically assessed with the Kruskal–Wallis test. To view the composition of the samples at each level of taxonomy, the QIIME2 taxa-bar-plot command was used.

Further statistical analyses were conducted using specialized software. PRIMER 7 software [36] was used to calculate a permutation-based multivariate analysis of variance (PERMANOVA) and to determine whether there were significant differences among predefined groups of samples (i.e., grouped according to the taxonomy of the host or its developmental stage). The same software was used to calculate a two-dimensional principal-coordinate analysis (PCoA) and non-metric multidimensional scaling (nMDS), as well as to generate PCoA and nMDS plots. In PAST 4.0 software [37] we performed similarity-percentage analysis (SIMPER) to calculate the average dissimilarities in bacterial-community structures between samples at the family level. According to the Diss/SD values, we identified those families which were primarily responsible for the observed differences among profiles (larger number means more consistent contributions to the dissimilarity between profiles). A similarity-profile (SIMPROF) test was used to identify well-defined groups of samples [38]. Finally, to illustrate the most abundant bacterial families and microbiome relationships across the tested samples, a heatmap and dendrogram were generated with the Bray–Curtis dissimilarity index. All statistical multivariate analyses were performed using PRIMER 7 software.

## 3. Results

### 3.1. Phylogenetic Analyses

Both mitochondrial and nuclear markers were successfully amplified for collected individuals. Seven COI haplotypes were identified based on the nucleotide substitutions present in the analyzed sequences (589 bp). In turn, twelve unique sequences were identified both for 28S (705 bp) and for ITS2 (828 bp) (Table 2). In those sequences, both nucleotide substitutions and indels were noticed. None of the identified haplotypes (neither mitochondrial nor nuclear) was shared between representatives of *H. leucanthemi* and *H. niger*.

**Figure 1:**
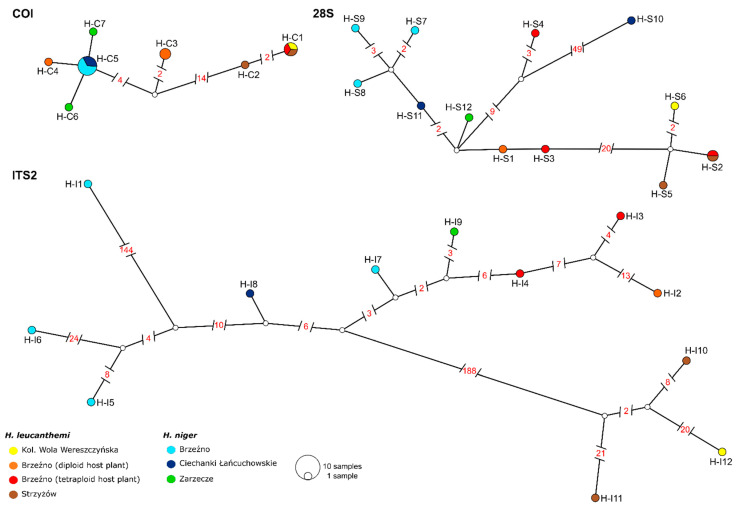
TCS networks resolving relationships among haplotypes identified in tested samples of *Haplothrips leucanthemi* and *H. niger*. Colored circles represent unique haplotypes encountered in analyses and lines connecting circles represent base-pair differences between haplotypes (values above 1 are shown). White circles represent intermediate haplotypes that were not encountered in the sampling. Size of the circle represents the frequency of the haplotype.

The topologies of the haplotype networks determined for selected markers did not support the distinctiveness of *H. leucanthemi* and *H. niger*, and the haplotypes identified for both thrips species did not form separate haplogroups (Figure 1). Complex relationships among identified haplotypes were also seen in the calculated values of the *p*-distance for pairs of those haplotypes (Table S2). In the case of COI, the values of the *p*-distance were estimated to be in the range of 0.00170–0.03565. The lowest values were reported for two pairs of haplotypes identified for *H. niger* (H-C5–H.C6 and H-C5–H-C7) but also for a pair of haplotypes determined for *H. niger* and *H. leucanthemi* (H-C5 and H-C4). In turn, the highest values were mainly reported for pairs including haplotypes determined for two selected species (i.e., pairs H-C1–H-C6, and H-C1–H-C7) but also for pair H-C1–H-C4 where both haplotypes were identified for *H. leucanthemi*. For nuclear markers, the values of the *p*-distance were estimated to be in the range of 0.00144–0.11705 for 28S and 0.00783–0.42629 for ITS2 (Table S2). Similar to the *p*-distance estimated for COI, the highest values were calculated for pairs including haplotypes that were determined for two thrips species.

**Figure 2:**
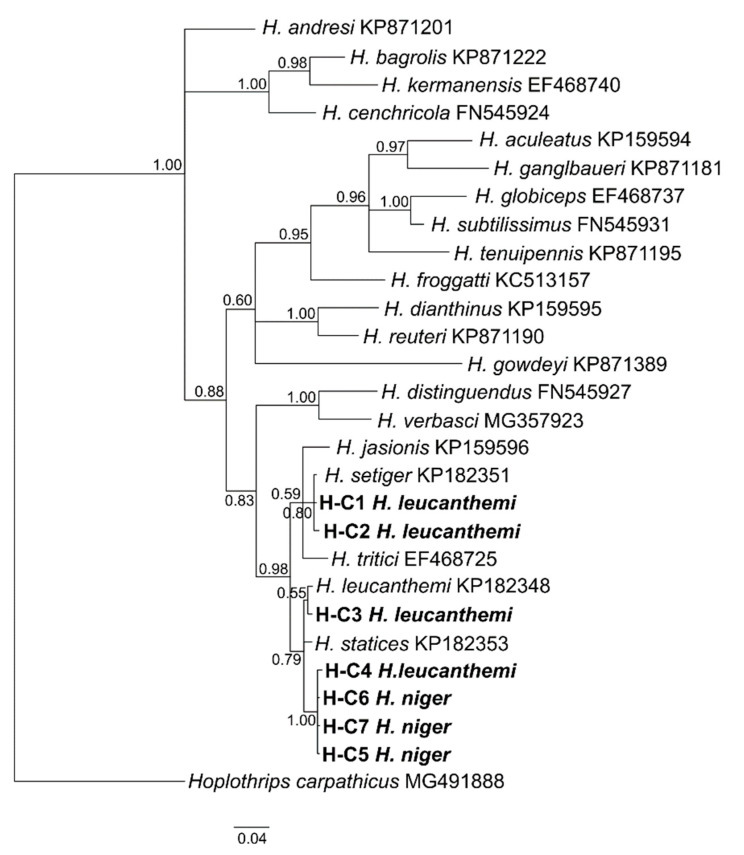
Bayesian dendrogram showing relationships among COI haplotypes identified in tested samples of *Haplothrips leucanthemi* and *H. niger*, and haplotypes determined for congeneric species. Posterior probability (PP) values are shown at nodes.

The mitochondrial COI haplotypes determined here were also used to resolve phylogenetic relationships among *H. leucanthemi*, *H. niger*, and other congeneric species (Figure 2). Neither *H. leucanthemi* nor *H. niger* formed a monophyletic clade. Haplotypes H-C1 and H-C2 (both determined for *H. leucanthemi*) were more similar to the sequence determined for *H. setiger* (GenBank accession number: KP182351) than to other haplotypes of *H. leucanthemi*. In turn, the haplotypes determined for *H. niger* were closely related to haplotype H-C4 that was identified for *H. leucanthemi,* which was also seen in the haplotype network (Figure 1). Surprisingly, the haplotype determined for *H. statices* (GenBank accession number: KP182353) was intermingled with those identified for both *H. leucanthemi* and *H. niger*. Additionally, values of the *p*-distance indicated the high similarity of those haplotypes (mean *p*-distance: 0.0174 between *H. leucanthemi* and *H. statices*, and 0.0150 between *H. niger* and *H. statices*) (Table S3).

### 3.2. Microbiota Profiling

After quality control and removing both mitochondrial and chloroplast sequences, we obtained a total of 1,359,460 demultiplexed sequences from 20 specimens of *H. leucanthemi* and *H. niger*. The mean sequence frequency was 69,973.0 per specimen (in the range of 21,193 for HN-C.1 to 95,998 for HL-B2.1). Analyses of alpha-diversity metrics revealed that the only significant difference was observed between the Shannon indices calculated for profiles determined for the *H. leucanthemi* adults and *H. niger* larvae (Kruskal–Wallis test (H): 4.20, *p* < 0.05).

**Figure 3:**
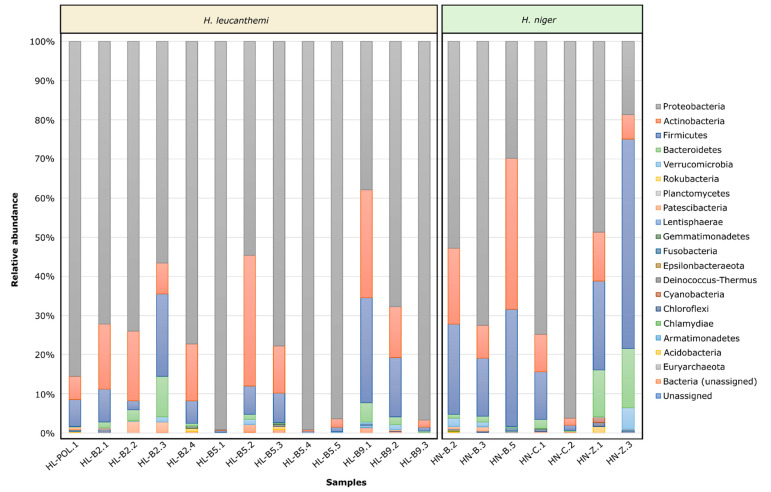
Phyla identified in profiles of microbial communities associated with tested *Haplothrips leucanthemi* and *H. niger* individuals.

The taxonomic classification yielded 19 phyla present in the analyzed microbial communities (Table S4). All tested samples contained high abundances of Proteobacteria, Actinobacteria, Firmicutes, and Bacteroidetes (Figure 3). Representatives of those phyla jointly accounted for more than 93.62% of the total identified microorganisms (in the range of 93.62% for HN-Z3 to 99.99% for HL-B5.1). In turn, Actinobacteria and Gammaproteobacteria were the most abundant groups at the class level, and Pseudomonadales, Enterobacteriales, and Rickettsiales were the most abundant orders. At the family level, Pseudomonadaceae, Rickettsiaceae, and Enterobacteriaceae (Table S4) were found with the highest average abundances. Moreover, two known endosymbiotic bacteria, *Wolbachia* and *Rickettsia,* were found in the analyzed profiles. They were identified mostly in bacterial communities associated with *H. leucanthemi* (*Wolbachia* in HL-B5.1, HL-B5.3, and HL-B9.2; *Rickettsia* in HL-POL.1, HL-B2.1, HL-B2.2, HL-B2.4, HL-B5.1, HL-B5.3, HL-B5.5, and HN-B2) (Table S4).

**Figure 4:**
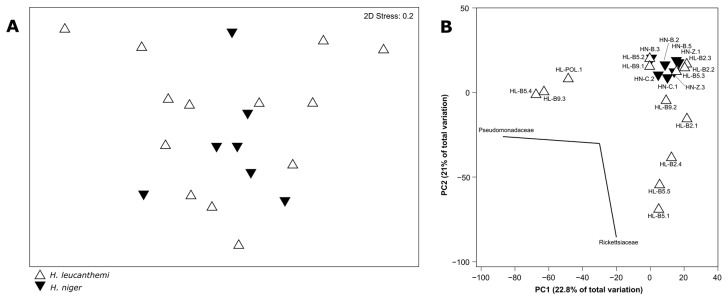
Similarity at the family level among tested microbiota profiles grouped according to the host taxonomy. (**A**)—non-metric multidimensional scaling (nMDS) ordination of *Haplothrips leucanthemi* and *H. niger* microbial communities; (**B**)—principal-coordinate analysis (PCoA) showing a two-dimensional ordination of tested microbiota profiles.

The nMDS analysis based on the abundances of all the identified microbial families showed a high similarity of the tested microbiota profiles that were grouped according to the taxonomy of the host (stress value = 0.2; Figure 4A). Although the results of the PERMANOVA analysis showed that the differences between those two groups of microbiota profiles are significant (Pseudo-*F* = 1.8953, *p* = 0.048), the *p*-value was close to the significance limit. In turn, the PERMANOVA analysis that was performed on datasets that were grouped according to both the taxonomy of host and its developmental stage showed significant differences among the groups (Pseudo-*F* = 1.7240, *p* = 0.024). Nevertheless, the pairwise tests showed that only the differences identified between the determined profiles for both *H. leucanthemi* and *H. niger* larvae are significant (Pseudo-*F* = 1.3413, *p* = 0.037). The differences among the profiles that were grouped according to the host plant were not significant (Pseudo-*F* = 1.3087, *p* = 0.158).

**Figure 5:**
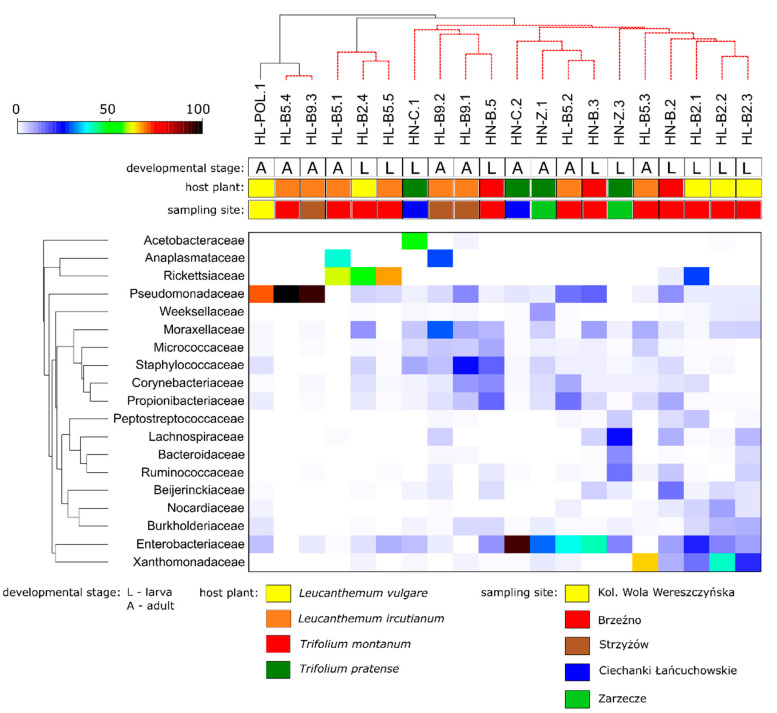
The heatmap showing relationships among tested profiles of bacterial communities associated with *Haplothrips leucanthemi* and *H. niger*. Only those families which were primarily responsible for the observed differences among samples were considered.

The high similarity of the determined microbiota profiles was also observed in the PCoA plot (Figure 4B). The analysis identified two families (Pseudomonadaceae and Rickettsiaceae) that were determined as grouping vectors for the tested profiles. In turn, the SIMPER analysis indicated that 19 families were primarily responsible for the differences among the samples. The relative abundances of these families were used to generate a heatmap for all the tested profiles (Figure 5). The SIMPROF analysis did not support the profiles that were grouped according to the taxonomy of the specimens for which these profiles were determined, the host’s developmental stage, the host plant, or the sampling site. However, in this case, the PERMANOVA analysis also supported significant differences between the groups that were determined according to the host’s taxonomy (Pseudo-*F* = 1.9760, *p* = 0.048), as well as both the host’s taxonomy and developmental stage (Pseudo-*F* = 1.7609, *p* = 0.027). The pairwise tests showed that the differences were significant between the profiles associated with *H. leucanthemi* and *H. niger* larvae (Pseudo-*F* = 1.3292, *p* = 0.047). Other potential factors did not cause significant differences among the tested microbial communities.

## 4. Discussion

Thysanoptera is one of the insect orders in which taxonomic ambiguities are frequently observed. Undoubtedly, reported problems are strictly correlated with the small body size of both immature and adult thrips, their morphological polymorphisms and frequent lack of solid morphological characteristics, the co-existence of different species on the same host plant, and the high intraspecific variation found within thrips populations [39,40]. Thus, unambiguous delimitation of thrips species based only on diagnostic morphological traits is often difficult and burdened with a risk of error for inexperienced entomologists. In those cases, additional analyses (e.g., analyses of molecular markers or ecological factors) seem to be very helpful. An integrative approach supported by molecular analyses of COI mitochondrial markers allowed us to, e.g., consider *Aeolothrips intermedius*, *Thrips tabaci* and *T. palmi* complexes of cryptic (sub)species, as well as to identify *Frankliniella occidentalis* and *Scirtothrips dorsalis* species [41,42,43].

In the present study, we applied molecular analyses to resolve the relationships between *Haplothrips leucanthemi* and *H. niger,* the latter of which is considered to be a parthenogenetic form and potential biotype of *H. leucanthemi*. Those analyses represent the first integrative approach to resolve the relationships between these species and to test the potential differences at both the mitochondrial and nuclear level, as well as in profiles of their associated microbiota, which could be congruent with the observed subtle morphological differences that have been previously noticed in both selected species.

The molecular analyses were primarily based on the divergence of the COI gene fragment. Previously, this mitochondrial marker has been successfully utilized in the molecular identification of thrips species (also those infesting economically important crops) (e.g., [7,44,45,46,47]), in the substantiation of morphological-characteristic-based species identification [48], and in the discrimination of thrips reproductive forms [49], as well as in the identification of genetic variants, biotypes, ecotypes, cryptic species and species complexes [42,50]. The idea of a COI-based species identification assumes that the intraspecific divergence in this barcode marker is lower than 2% [51,52]. However, for several thrips species, the COI sequences demonstrate high intraspecific diversity resulting in a low barcode gap among those species [53]. Our results are in line with that observation (the highest value of the *p*-distance between two COI haplotypes determined for *H. leucanthemi* was 3.6%). In turn, the lowest value of the *p*-distance that was calculated for haplotypes determined for *H. leucanthemi* and *H. niger* was 0.02%, and such a value is characteristic of intraspecific rather than interspecific divergence. Moreover, the analysis of the mean *p*-distances that were calculated for the COI data determined for other *Haplothrips* species revealed that the low barcode gap is also observed in this genus (e.g., 1.7% between *H. leucanthemi* and *H. statices*, and 1.5% between *H. niger* and *H. statices*). Nevertheless, the analyses based on the COI revealed that the relationships between *H. leucanthemi* and *H. niger* are vague, and that specimens identified as *H. niger* should instead be recognized as *H. leucanthemi*. That observation was also supported by the analyses of nuclear markers.

The topology of the networks based on 28S and ITS2 data also did not reveal the clear separation of those two thrips representatives.

As an additional part of our chosen integrative approach, we also determined and analyzed the microbiota profiles associated with the collected specimens. The obtained results were congruent with those previously described for other thrips species. Proteobacteria, Actinobacteria, Firmicutes, and Bacteroidetes were found to be the most abundant bacterial phyla. Those groups are dominant in microbiota profiles of different insect species [54,55,56], and were also the most abundant in bacterial communities associated with other thrips, e.g., *Hoplothrips carpathicus* [57], *Scirtothrips dorsalis* [58], *Thrips palmi* [56], and *T. tabaci* [59].

Further analyses were performed to identify potential differences among the profiles determined for both *H. leucanthemi* and *H. niger* specimens. However, the nMDS, PCoA, and SIMPROF analyses showed a high similarity of the tested bacterial communities. Surprisingly, the SIMPROF analysis did not support the groups of profiles that were identified according to the developmental stage of the host.

Differences in the associated microbiota, which are congruent with the host’s development, have been previously reported for the fungivorous thrips *Hoplothrips carpathicus* [57] and other insect species (e.g., the oriental fruit flies *Bactrocera dorsalis* [60] and *B. tryoni* [61], the stag beetle *Phalacrognathus muelleri* [62], or the fungivorous beetle *Bolitophagus reticulatus* [63]), where significant dissimilarities were especially observed between the microbiota associated with larvae and adults. In the present study, the sample size was limited and additional microbiota profiles associated with those two developmental stages of *H. leucanthemi* and *H. niger* should be analyzed to confirm the lack of significant differences among their associated bacterial communities (especially taking into account that the results of the pairwise PERMANOVA tests supported the significant differences between the profiles associated with *H. leucanthemi* larvae and adults, and between the profiles determined for both *H. leucanthemi* and *H. niger* larvae).

Surprisingly, the results of the PERMANOVA analysis supported the significant differences between the microbiota profiles that were grouped according to the host taxonomy. Previous studies on insect microbiota have shown that the host’s taxonomy significantly affects gut bacterial communities [55,56]. Nevertheless, the *p*-value calculated here was on the verge of significance, so the observed differences are subtle rather than diagnostic.

The microbiota profiling performed in the present study revealed the presence of two known endosymbiotic bacteria (i.e., *Wolbachia* and *Rickettsia*) in the tested bacterial communities. Both of these endosymbionts have been described as bacteria that are able to manipulate host’s reproduction in several ways in order to enhance their own spread, and the induction of parthenogenesis is one such modification [64]. Endosymbiont-induced parthenogenesis has been observed in different hosts, e.g., mites, wasps, and thrips [64]. Moreover, further antibiotic treatment of thrips, e.g., *Franklinothrips vespiformis* or *Hercinothrips femoralis,* were introduced the production of males that copulated with females [65,66]. However, in the case of *F. vespiformis*, the experimental mating under laboratory conditions did not affect the sex ratios of the next generation, suggesting that the sperm do not fertilize the eggs [66]. *H. niger* is widely recognized as a parthenogenetic form of *H. leucanthemi*. Thus, one may expect that *Wolbachia* and *Rickettsia* should be present in the bacterial communities that are associated with its larvae and adults, but those bacteria were mostly observed in *H. leucanthemi* microbiota and were identified only in one *H. niger* profile. Thus, the endosymbiont-induced parthenogenesis in *H. niger* seems less likely. However, the present study should be considered as preliminary research, as a limited number of specimens were included in the main analyses. Thus, this study should be continued in a more comprehensive approach to confirm the obtained results.

## 5. Conclusions

An integrative approach based on the analyses of mitochondrial and nuclear molecular markers and microbiota profiling was used to determine the relationships between *H. leucanthemi* and *H. niger*. The results of the molecular analyses revealed the high intraspecific diversity of *H. leucanthemi* and did not support the distinctiveness of *H. niger*. The identified microbiota profiles were similar in both species and the performed analyses did not support the distinctiveness of *H. niger*. Significant differences were, in turn, observed between *H. leucanthemi* and *H. niger* larvae. Moreover, two known endosymbiotic bacteria were found in the analyzed microbiota profiles (i.e., *Wolbachia* and *Rickettsia*). Nevertheless, those symbionts were not predominantly found in the bacterial communities that are associated with *H. niger* and thus, its impact on the parthenogenetic mode of its reproduction seems less likely. However, in the present preliminary study, a limited number of *H. leucanthemi* and *H. niger* specimens were tested and further, more comprehensive studies are needed to confirm the obtained results.

## Figures and Tables

**Table 1:** Detailed information about specimens of both *Haplothrips leucanthemi* and *H. niger* collected from selected sampling sites for further molecular analyses.

Species	Sample Code	Locality/Coordinates	Host Plant	Collected Developmental Stages
*Haplothrips leucanthemi*	HL-POL	Kol. Wola Wereszczyńska 51.45° N, 23.15° E	*Leucanthemum vulgare* (diploid)	HL-POL.1—adult
	HL-B2	Brzeźno 51.17° N, 23.62° E		HL-B2.1—larva
				HL-B2.2—larva
				HL-B2.3—larva
				HL-B2.4—larva
	HL-B5	Brzeźno 51.17° N, 23.62° E	*Leucanthemum ircutianum* (tetraploid)	HL-B5.1—adult
				HL-B5.2—adult
				HL-B5.3—adult
				HL-B5.4—adult
				HL-B5.5—adult
	HL-B9	Strzyżów 50.85° N, 24.02° E		HL-B9.1—adult
				HL-B9.2—adult
				HL-B9.3—adult
*Haplothrips niger*	HN-B	Brzeźno 51.17° N 23.62° E	*Trifolium montanum*	HN-B.2—larva
				HN-B.2—larva
				HN-B.5—larva
	HN-C	Ciechanki Łańcuchowskie 51.28° N, 22.95° E	*Trifolium pratense*	HN-C.1—larva
				HN-C.2—adult
	HN-Z	Zarzecze near San River 50.53° N, 22.20° E		HN-Z.1—adult
				HN-Z.3—larva

**Table 2:** Distribution of mitochondrial and nuclear haplotypes identified among sequences obtained for tested *H. leucanthemi* and *H. niger* individuals. Numbers in brackets indicate numbers of sequences identified as particular haplotypes.

Sample	COI	28S	ITS2
*H. leucanthemi*			
Kol. Wola Wereszczyńska (HL-POL)	H-C1 (1)	H-S6 (1)	H-I12 (1)
Brzeźno—diploid host plant (HL-B2)	H-C3 (2), H-C4 (1)	H-S1 (1)	H-I2 (1)
Brzeźno—tetraploid host plant (HL-B5)	H-C1 (1)	H-S2 (1), H-S3 (1), H-S4 (1)	H-I3 (1), H-I4 (1)
Strzyżów (HL-B9)	H-C1 (1), H-C2 (1)	H-S2 (1), H-S5 (1)	H-I10 (1), H-I11 (1)
*H. niger*			
Brzeźno (HN-B)	H-C5 (4)	H-S7 (1), H-S8 (1), H-S9 (1)	H-I1 (1), H-I5 (1), H-I6 (1)
Ciechanki Łańcuchowskie (HN-C)	H-C5 (2)	H-S10 (1), H-S11 (1)	H-I7 (1), H-I8 (1)
Zarzecze near San River (HN-Z)	H-C6 (1), H-C7 (1)	H-S12 (1)	H-I9 (1)

## Data Availability

All newly obtained sequences are deposited in the GenBank database (accession nos. MZ047773-MZ047779, MZ191780-MZ19179, and MZ478147-MZ478157). Raw NGS data are deposited and fully available in the European Nucleotide Archive (accession no. PRJNA730011).

## Supplementary Materials

The following are available online at https://www.mdpi.com/article/10.3390/insects13030279/s1, Table S1: Thermocycling conditions for a routine amplification of selected mitochondrial and nuclear markers, Table S2: Values of *p*-distance and standard error for mitochondrial and nuclear haplotypes identified for *Haplothrips leucanthemi* and *H. niger*, Table S3: Mean values of *p*-distance and standard error for mitochondrial COI haplotypes identified for *Haplothrips* species. COI sequence for *Thrips tabaci* was used as an outgroup, Table S4: The relative abundance of microbial 16S rDNA sequences at different taxonomic levels.

## Appendix A

*Sample Collection for Morphometric Study*

The morphometric study on larvae of the two selected species were conducted in 2010–2011. The specimens of *H. leucanthemi* and *H. niger* were collected at various sites in eastern and southern Poland. Archival specimens deposited at the Department of Zoology and Nature Protection, Maria Curie-Skłodowska University in Lublin were also used in the study (Table A1). Most of the insects (except those donated by Zawirska) were collected in the field mainly along the roadsides and on meadows where their host plants grow. The insects were reared under the laboratory conditions which were described by Kucharczyk and Stanisławek [16]. Finally, the microscopic slides have been made in accordance with the procedures described by Mound and Kibby [67]. The morphometric characteristics of 15–20 specimens of larvae collected on each sampling site (Table A1) were analyzed. Photos were taken using an OLYMPUS BX 61 microscope and an Olympus CellSens Dimension image analyzer.

*Morphological Comparison*

Both adults and larvae of *H. leucanthemi* and *H. niger* have been collected in different regions of Poland (Table A1). The adults differed in a few characteristics such as the length of prothorax and abdominal segment IX, which are longer in *H. leucanthemi*, and prothorax epimeral setae which in turn are longer in *H. niger*. Moreover, the postocular setae are pointed at the apex of the former and blunt in the latter species. Our observations confirmed data included in the keys of Schliephake and Klimt [14]. The new feature observed by the authors in collected adult specimens of both species was the size of spiracle on VIII abdominal segment. The spiracle is round and its’ diameter is larger (15–20, most often 17,5 µm) in *H. leucanthemi* than in *H. niger* (10 µm).

Measurements of the body and chaetotaxy of the head, thorax and dorsal and ventral side of abdomen are given for second instar larvae of both species. Analysis of the morphological characteristics shows some differences in the body length, antennae length, length of prothorax setae D1, D4, D5 and D6. These values are higher in *H. niger* while both the length and width of spiracles on mesothorax and abdomen (segments II and VIII), and the numbers of cells in spiracles are higher in *H. leucanthemi* larvae (Table A2, Figure A1, Figure A2 and Figure A3).

**Table A1:** Detailed information about sampling sites of archival specimens of both *Haplothrips leucanthemi* and *H. niger* used for further morphometric analyses.

Species	Locality/Coordinates	Host Plant	Developmental Stage	Date and Author of Collected Materials
*Haplothrips leucanthemi*	Rowokół near. Smołdzino N 54.66 E 17.21	*Chrysanthemum leucanthemum*	Second larval instar	19.07.2000 I. Zawirska
	Urszulin N 51.40 E 23.20			07.06.2010 K. Stanisławek
	Zawoja N 49.67 E 19.57			19.07.2010 A. Dembicka
	Zastawie N 51.39 E 23.27			15.06.2010 K. Stanisławek
	Białka (near Łęczna) N 51.54 E 23.01			23.05.2011 K. Stanisławek
	Ciechanki Łańcuchowskie N 51.28 E 22.95			23.05.2011 K. Stanisławek
	Jaszczów Kolonia N 51.21 E 22.95			23.05.2011 K. Stanisławek
	Werbkowice N 50.75 E 23.78			30.05.2011 M. Kucharczyk
	Udrycze (near Zamość) N 50.80 E 23.28			20.05.2011 K. Stanisławek
	Wojtkowa (Bieszczady Mts.) N 49.57 E 22.55			13.08.2011 K. Stanisławek
	Dziewięcierz N 50.31 E 23.42			25.06.2011 K. Stanisławek
	Mała Rawka (Bieszczady Mts.) N 49.11 E 22.57			04.08.2011 H. Kucharczyk
*Haplothrips niger*	Ursynów (Warszawa) N 52.15 E 21.03	*Trifolium pratense*	Second larval instar	22.10.1984 I. Zawirska
	Warszawa, Huta N 52.30 E 20.91			27.06.1995 I. Zawirska
	Jakubowice Konińskie N 51.31 E 22.55			13.06.2010 K. Stanisławek
	Lublin N 51.25 E 22.57			25.05.2011 20.06.2011 22.06.2011 K. Stanisławek
	Białka (near Łęczna) N 51.54 E 23.01			23.05.2011 K. Stanisławek
	Rudnik (near Lublin) N 51.28 E 22.60			20.06.2011 K. Stanisławek
	Ciechanki Łańcuchowskie N 51.28 E 22.95			23.05.2011 K. Stanisławek
	Polanka Horyniecka (Roztocze) N 51.25 E 22.57			25.06.2011 K. Stanisławek
	Bandrów Narodowy (Bieszczady Mts.) N 49.39 E 22.70			13.08.2011 K. Stanisławek
	Muczne (Bieszczady Mts.) N 49.13 E 22.74	*Trifolium repens*	Second larval instar	14.08.2011 K. Stanisławek

**Table A2:** Character states used in morphological analyses of *Haplothrips leucanthemi* and *H. niger* second larval instar with the range of obtained measurements. Values are given in µm. The mean or the modal values (number of facets) are given in brackets.

Character	*H. leucanthemi*	*H. niger*
Body length	1200–1780 (1422)	1317–1933 (1636)
Head—length/width	190–219 (206.5)/110–153 (133.0)	185–211 (197.0)/109–134 (120.5)
Head—length/width proportion	1.5–1.9 (1.65)	1.4–1.8 (1.63)
Head—length of dorsal setae D1, D2	D1 37.5–51.0 (43.2) D2 14.0–22.5 (17.1)	D1 36.0–51.0 (44.2) D2 12.0–20.0 (16.9)
Length of antennae	206–250 (223.5)	214–272 (248.0)
Prothorax—length of dorsal setae D1, D4, D5, D6	D1 22.0–32.0 (27.2) D4 28.0–43.0 (35.2) D5 53.0–69.0 (60.5) D6 60.5–77.0 (68.7)	D1 24.0–38.0 (31.3) D4 30.0–44.0 (37.5) D5 51.0–74.0 (65.3) D6 61.5–85.0 (73.3)
Mesothrax—length of dorsal setae D1, D2, D3, D5, D6	D1 26.0–38.0 (31.8) D2 16.0–22.5 (18.4) D3 19.0–30.0 (22.8) D5 55.0–77.0 (64.0) D6 35.0–50.0 (42.1)	D1 29.0–41.0 (35.6) D2 17.0–26.0 (22.5) D3 18.0–29.0 (25.7) D5 62.0–81.0 (72.5) D6 36.0–49.0 (42.7)
Metathorax—length of dorsal setae D1, D2, D3, D5, D6	D1 27.0–40.5 (33.6) D2 16.0–24.0 (19.8) D3 17.5–37.5 (27.1) D5 59.0–75.0 (66.6) D6 32.5–52.0 (41.8)	D1 31.0–44.0 (38.1) D2 18.0–25.0 (21.7) D3 19.0–35.0 (27.3) D5 64.0–80.0 (73.2) D6 38.0–50.0 (44.7)
Abdominal tergite VIII—length of dorsal setae D1, D2	D1 45.0–62.5 (53.1) D2 55.0–70.0 (61.0)	D1—52.0–72.0 (62.5) D2—52.0–72.0 (63.1)
Abdominal tergite IX—length of dorsal setae D1, D2	D1 72.5–90.0 (81.1) D2 39.0–53.5 (45.2)	D1 72.0–91.0 (82.5) D2 32.0–51.0 (42.7)
Abdominal sternite VIII—length of ventral setae V1, V2, V3	V1 57.0–88.0 (72.0) V2 12.0–21.0 (16.1) V3 46.0–68.5 (56.3)	V1 61.0–90.0 (75.1) V2 11.0–20.0 (16.0) V3 52.0–67.0 (59.9)
Abdominal sternite IX—length of ventral setae V1, V2	V1 73.0–102.0 (86.1) V2 42.5–60.0 (50.1)	V1 70.0–99.0 (85.5) V2 32.0–56.0 (46.3)
Abdominal segment X—length	69.0–89.0 (78.6)	67.0–84.0 (77.1)
Abdominal segment X—proportion of anterior and posteriori rand width	2.1–3.3 (2.8)	2.1–2.6 (2.4)
Abdominal segment XI, length of setae D1, V1, V2	D1 17.5–28.0 (22.4) V1 10.5–20.0 (14.2) V2 122.0–160.0 (138.0)	D1 18.0–28.0 (23.8) V1 9.0–18.0 (14.1) V2 118.0–170.0 (147.0)
Meso- and metanotum—sclerotization at basis of setae	weak or absent, may be stronger around setae D3, D5, D6	weak or absent
Abdomen—sclerotization at basis of dorsal setae	weak or absent, may be stronger around setae on segments VI-VIII	weak or absent
Spiracle on mesonotum—length/width	20–28 (23.8)/37–47 (42.1)	19–21 (20.2)/35–38 (36.3)
Spiracle on mesonotum—number of facets (cells). Mean number of cells	27–46 (35) >30	20–27 (23) <30
Spiracles on abdominal tergite II—length/width	14–26 (18)/17–27 (22)	14–16 (15)/16–21 (19)
Spiracles on abdominal tergite II—number of facets (cells)	7–22 (15)	6–17 (11)
Spiracles on abdominal tergite VIII—diameter	17–27 (22)	16–23 (18)
Spiracles on abdominal tergite VIII—number of facets (cells) Mean number of cells	20–30 (24) >15	10–15 (12) <15
