# Supplementary material for: Integrative Insight into Relationships between Florivorous Thrips Haplothrips leucanthemi and H. niger (Insecta, Thysanoptera)
Source: Insects. 2022 Mar 11;13(3):279. doi: 10.3390/insects13030279 (PMC8950692; doi:10.3390/insects13030279)
Supplement: Supplementary file 1 [file insects-13-00279-s001.zip › Table S1.pdf]

Table S1. Thermocycling conditions for a routine amplification of selected mitochondrial and nuclear markers.

	**COI**	**28S**	**ITS2**
initial denaturation	94°C / 5 min	98°C / 1 min	94°C / 2 min
cycles	94°C / 1 min<br>51.2°C / 1 min<br>72°C / 1 min<br>35 cycles	98°C / 10 sec<br>45.7°C / 10 sec<br>72°C / 45 sec<br>35 cycles	94°C / 15 sec<br>51.9°C / 15 sec<br>68°C / 30 sec<br>35 cycles
final extension	72°C / 5 min	72°C / 5 min	68°C / 5 min
polymerase	JumpStart Taq ReadyMix, Sigma-Aldrich, Germany	Platinum SuperFi Green PCR Master Mix, Invitrogen, Thermo Fisher Scientific, USA	Platinum II Hot-Start Green PCR Master Mix, Invitrogen, Thermo Fisher Scientific, USA
